# Measurement of cardiac troponins to detect myocardial infarction using high-sensitivity assays: South African guidelines

**Published:** 2012-11

**Authors:** Rhena Delport, James A Ker

**Affiliations:** Department of Chemical Pathology, School of Medicine, Faculty of Health Sciences, University of Pretoria, Pretoria, South Africa; Department of Internal Medicine, School of Medicine, Faculty of Health Sciences, University of Pretoria, Pretoria, South Africa

With the use of specific cardiac markers with higher sensitivity, new perspectives have emerged on the nature of myocardial necrosis and injury, which are associated with acute coronary syndrome (ACS). The third universal definition of myocardial infarction^1^ now classifies myocardial infarction (MI), based on the relevant pathology, clinical presentation, prognosis and treatment strategy, as spontaneous MI (type 1), MI secondary to an ischaemic imbalance (type 2), cardiac death due to sudden fatal MI (type 3), and MI associated with revascularisation procedures (types 4 and 5). What has also become evident is the extent of necrosis and injury that is associated with pathologies of other organs and conditions.^1^-^3^

The clinical circumstances associated with elevated values of cardiac troponin (c-Tn) due to myocardial injury have been listed,^1^ and comprise conditions related to primary myocardial ischaemia, conditions related to supply/demand imbalance of myocardial ischaemia, conditions not related to myocardial ischaemia and conditions related to multi-factorial or indeterminate myocardial injury. A shift in focus is apparent, not only from valuing these highly sensitive cardiac biomarkers for their exceptional diagnostic sensitivity and negative predictive value for the diagnosis of MI, but also for their application in ACS risk stratification.^1^,^4^-^6^

Guidelines on the use of high-sensitivity cardiac troponin (hs-cT) markers have recently been set in the consensus statement of the Ethics and Guidelines Standing Committee of the South African Heart Association.^7^ This editorial aims to appraise these guidelines in the light of more recent research findings and newer guidelines.

The committee recommends that high-sensitivity troponin assays be widely adopted as the preferred biomarker for the diagnosis of myocardial infarction, based on evidence of earlier diagnosis of MI, more reliable ruling out of MI, and shortening of the chest pain triage (to four hours compared to former assays). All cardiac troponin measurements are to be reported in ng/l. The first sample is to be collected on first assessment, followed by a second sample after three hours, should the first value be lower than the 99th percentile (URL) of a normal reference population for the specific assay, or between the URL and the WHO-defined URL for MI. Serial measurements are to be reported as percentage change. A specific algorithm for both hs-cTropT and hs-cTropI is proposed for the diagnosis of MI.

The Expert Consensus document on the third universal definition of myocardial infarction^1^ states that sample repeat may be three to six hours later, followed by further sampling depending on uncertainty concerning timing of the initial symptoms and whether the injury was evolving or resolving.^4^,^8^,^9^ Rule-in for MI constitutes a rise and/or fall in values, with one value above the decision limit (99th percentile value), using an assay with an imprecision (coefficient of variation) ≤ 10% if accompanied by a strong pre-test likelihood, the diagnosis being based mainly on the latter.^1^ Repeat measurements display the dynamic pattern of troponin values and aid in differentiating between acute and chronic causes of troponin elevation in the circulation.^4^

The guidelines defined for South Africa (SA)^7^ differ from those in the Consensus document.^1^ They state: ‘The percentage change (rise or fall) in hs-cT levels in two samples three hours apart is used to establish a diagnosis of MI when the troponin level is below the WHO cut-off. For troponin I a 50% change in an initial value is diagnostic of MI. In the case of troponin T, a 50% change in an initial value of < 53, or a 20% change in an initial value between 53 and 100 ng/l, is diagnostic of MI.’ They are similar to those set by the study group on biomarkers in cardiology of the ESC Working Group on Acute Cardiac Care, in that the 50% change rule is applied for the second sample,^9^ but they do not apply the WHO cut-off point and state as prerequisite for rule-in that the values at three hours (and optionally at six hours) be greater than the URI.

In the South African guidelines,^7^ the WHO cut-off values are also taken into consideration for decision making, in that values between the URL and the WHO cut-off values are subject to repeat measurement at three hours, the percentage change being dependent on the first assessment value being smaller than the WHO cut-off values. Of note is that use of change as a measure for rule-in may increase the specificity for MI, but at the cost of a decrease in sensitivity,^9^-^11^ and that, as stated by Thygesen *et al.*,^1^ ‘It should be clear that dynamic changes are not specific for MI but rather are indicative of active myocardial injury with necrosis’.

The validity of the use of the URL^12^-^15^ as well as repeated measurements at three hours for rule-in or rule-out of MI^3^,^16^-^18^ have been substantiated in several studies. The selection strategy for the reference population, however, markedly influences the 99th percentile reference values for troponin assays if it does not consider relevant demographic, biological and clinical variables and this affects the diagnostic performance of highly sensitive immunoassays,^4^,^19^-^21^ as suggested in the SA guidelines.^7^

Furthermore, inter-assay differences concerning reference values for specific populations appear to impact on risk stratification.^22^-^24^ A higher cut-off point for the diagnosis on NSTEMI may be appropriate in patients with mildly elevated hs-TnI and without evidence for STEMI,^25^ and use of absolute change over serial measurements is suggested to perform better and decrease time to rule-in and rule-out of NSTEMI compared with relative change.^11^,^26^-^29^

The 20% limit of defining a significant increase from the time of first assessment if baseline values are above the URL has been established within the National Academy of Clinical Biochemistry laboratory medicine practice guidelines^30^ and represents a significant (> 3) standard deviation variation on the basis of a 5–7% analytical imprecision (analytical CV).^12^,^31^,^32^ This increment of increase has been proven to be clinically useful^8^,^32^-^35^ but is assay dependent,^16^,^17^,^36^-^38^ and remains a challenge that requires further clinical and prospective studies, as concluded by Lippi *et al.* and others.^9^,^40^ The 50% limit of defining a significant increase if baseline values are below the URL does not appear to be based on high-level evidence but purportedly optimises the overall accuracy of MI diagnosis.^9^,^11^,^27^

Other complexities of measurement, as eluded to by Thygesen,^9^ are the substantial differences between ‘high-sensitivity’ assays and the concern that the manufacturers’ claims for assay precision cannot be achieved in clinical laboratories. Relevant analytical issues alluded to in the SA guidelines are falsely high values because of heterophile antibodies and human auto-antibodies interfering with the assay,^40^-^42^ and falsely low levels with haemolysis.^43^,^44^

In addition, Lippi *et al.*^4^ reported interferences being observed, caused by rheumatoid factor, complement, presence of fibrin in serum or plasma after centrifugation of the sample, unsuitable samples (e.g. haemolysed, lipaemic, icteric), and analytical errors (e.g. instrument malfunctioning). Of interest is a report by Gould *et al*. on carry-over to subsequent samples with certain analysers, potentially leading to false-positive results.^45^

## Conclusion

The South African guidelines on the use of high-sensitivity cardiac troponins as biomarkers are timely and of great value, provided that clinicians take up the challenge of applying them clinically.
